# Factors Governing the Catalytic Insertion of CO_2_ into Arenes – A DFT Case Study for Pd and Pt Phosphane Sulfonamido Complexes

**DOI:** 10.1002/chem.202104375

**Published:** 2022-03-21

**Authors:** Markus Hölscher, Gregor Kemper, Sangeth Jenthra, Carsten Bolm, Walter Leitner

**Affiliations:** ^1^ Institut für Technische und Makromolekulare Chemie RWTH Aachen University Worringer Weg 2 52074 Aachen Germany; ^2^ Institut für Organische Chemie RWTH Aachen University Landoltweg 1 52074 Aachen Germany; ^3^ Max-Planck-Institut für Chemische Energiekonversion Stiftstraße 34–36 45470 Mülheim a. d. Ruhr Germany

**Keywords:** Catalysis, CO_2_, DFT, aromatic acids, Palladium, Platinum

## Abstract

The potential of Pd/Pt complexes for catalytic carboxylation of arenes with CO_2_ is investigated by means of computational chemistry. Recently we reported that the bis[(2‐methoxyphenyl)phosphino]‐benzenesulfonamido palladium complex **1** inserts CO_2_ reversibly in its Pd−C(aryl) bond generating carboxylato complex **2**. In the present work we study how geometric and electronic factors of various ligands and substrates influence the overall activation barrier (energy span, ES) of a potential catalytic cycle for arene carboxylation comprising this elementary step. The tendency of the key intermediates to dimerize and thus deactivating the potential catalysts is examined as well as the role of the base, which inevitably is needed to stabilize the reaction product. We show that Pd and Pt complexes **I(Pd)**‐**L16**‐**S1** and **I(Pt)**‐**L16**‐**S1** do not dimerize, enable the computation of complete catalytic cycles, and show interestingly low ES values of 26.8 and 24.5 kcal/mol, respectively.

## Graphical abstract

**Which catalyst does the job**? A DFT based catalyst screening with Pd/Pt complexes for the catalytic direct insertion of CO_2_ into the C−H bonds of arenes to provide a carbonic acid indicates that a catalytic process might well be achievable, when the multi‐parameter system is carefully tuned. Not only the choice of metal is important, also the size and shape as well as the electronic influences of the ligand and the electronic influences of the substrate needs to be concerned. The appropriate base also must be found and some undesired side reactions must be suppressed.

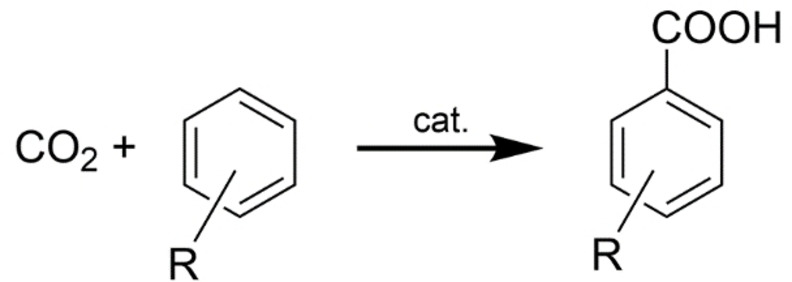

The utilization of carbon dioxide as a raw material in organic synthesis is a highly topical area in the field in organometallic catalysis.^[1]^ The synthesis of carboxylic acids (RCO_2_H) seems particularly attractive as the functional group can be constructed by incorporation of the entire CO_2_ molecule.^[2]^ Aromatic carboxylic acids (ArCO_2_H) are potential synthetic targets given their widespread application in polymers, fine and agro chemicals, as well as pharmaceuticals. Indeed, salicylic acid is produced by carboxylation of sodium phenolate with CO_2_ using the Kolbe‐Schmitt synthesis. However, just like with other standard carboxylation reactions involving, for example, Grignard reagents, the requirement of stoichiometric metallated substrates jeopardizes to a significant extend the benefits of introducing CO_2_ back into the carbon cycle.^[3]^ In this context, the direct catalytic carboxylation by insertion of CO_2_ into the C−H bonds of arenes remains an ongoing challenge, which has witnessed interesting developments, reported on, for instance, by the groups of Nolan,^[4]^ Iwasawa^[5]^ and Li.^[6]^ Computational activities in the field of relevant C−C bond formation using CO_2_ have been summarized recently by Hopmann and coworkers.^[7]^

In our activities targeted at the direct catalytic insertion of CO_2_ in arenes we have been studying catalyst systems that do not require any further reagents apart from the catalyst and the base necessary to create the thermodynamic driving force for the reaction. In close interplay between experiment and theory we initially have investigated ruthenium pincer complexes and revealed their potential and limitations for catalytic carboxylation.^[8]^ From a broad in silico screening of potential other lead structures, we selected Pd complexes with sulfonamide phosphane ligands as the subject for a more in‐depth analysis. Notably, we could demonstrate experimentally that CO_2_ inserts reversibly into the Pd‐aryl bond of **1** resulting in the carboxylato complex **2** (Scheme 1).^[9]^

**Scheme 1:**
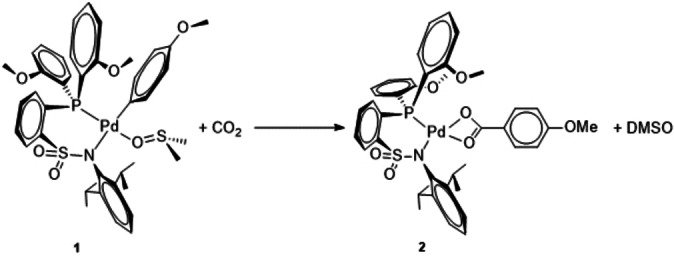
Insertion of CO_2_ into the Pd−C(aryl) bond of **1** forming complex **2**, which contains a metal‐bound carboxylate.[9]

The carboxylato complex **2** constitutes the central intermediate of type **I** on the potential energy surface of a catalytic carboxylation of arenes as shown in Figure 1. De‐coordination of the κ^2^‐carboxylate on either side *trans* to the bidentate P−X ligand opens to isomeric pathways **II** to **VI** or **IIa** to **VIa**. The experimentally verified insertion reaction shown in Scheme 1 corresponds to the elementary step from **V** to **I** via **VI** (Figure 1). The experimental study also revealed a tendency of complexes **I** to form carboxylate‐bridged dimers of type **[I_2_]** as potential off‐loop species which are very stable and therefore increase the energy span (ES; overall activation barrier of the reaction) to unfavorably high values.

**Figure 1:**
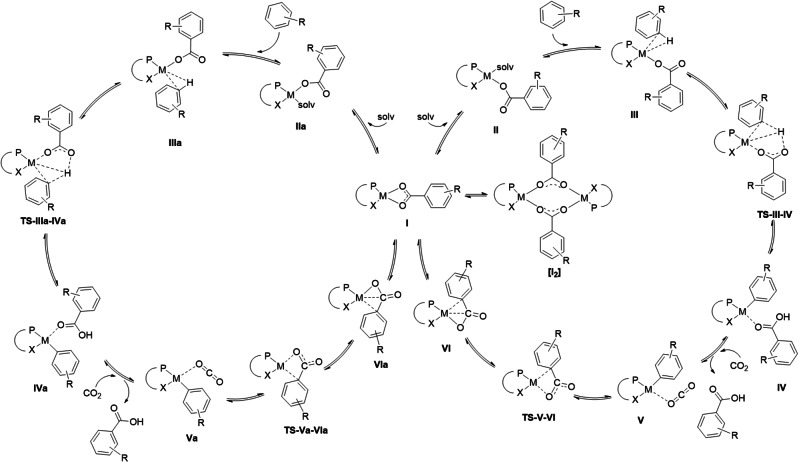
Generalized catalytic cycle for the direct insertion of CO_2_ into arenes computed for concrete Pd and Pt complexes in this work with DFT methods. (see below).

In the present study, we used DFT calculations to explore the potential of complexes of type **I** as crucial intermediates for the direct carboxylation or arenes on basis of structure/reactivity relationships on the catalytic network shown in Figure 1. The geometry optimizations and energy computations in this work were carried out in condensed phase using the experimentally verified solvent dichloromethane with the B97D3‐BJ density functional and the def2‐TZVP(ECP)‐ or def2‐SVP(ECP)‐ basis sets employing the IEFPCM‐SMD solvent/radii model (all computational details, see Supporting Information).^[10]^ While B97D3‐BJ mainly was chosen for reasons of computational efficiency comparisons with other density functionals were made to some extent for the critical intermediates (TDI) and transition states (TDTS; details see Supporting Information).

It should be noted at this stage that for DFT‐screening purposes it is not necessary to compute the complete catalytic cycle for each and every substrate or ligand variation. According to the energy profile computed in earlier work,^[9]^ the relative energies of the lowest energy intermediate **I**–**L3** (turn over determining intermediate, TDI) as well as the highest transition state **TS**‐**V**–**VI**‐**L3** (turn over determining transition state, TDTS) are separated energetically clearly from all other intermediates and transition states. Consequently, these two stationary points (i.e. the reference point **I** as well as transition state **TS**‐**V**–**VI**) can be chosen for screening purposes to compute their structures/energies as proxy for the energy difference comprising the ES. Complete catalytic cycles can then be computed for selected complexes following this initial screening.

In a systematic analysis, we will discuss important aspects in sections 1–5, resulting in the computation of cycles for selected catalyst systems (section 6), which provides valuable information for future experimental work. The sections are concerned with the following aspects:

1. The tendency of carboxylate complexes like **2** (resembling **I** in Figure 1) to dimerize to very stable complexes **[I_2_]**.
2. The influence of variations in the structure of the substrate.
3. The influence of variations in the structure of the ligand.
4. The influence of combined variations in the substrate and the ligand.
5. The base, which inevitably is needed to stabilize the endergonic reaction product (the carbonic acid).
6. Identifying from 1–5 a concrete Pd catalyst system for the computation of complete catalytic cycles necessary to evaluate the ES of such a cycle and extension of the analysis to the corresponding Pt‐catalyst for comparison.

## 1. Dimerization of complexes of type I

**Figure 2:**
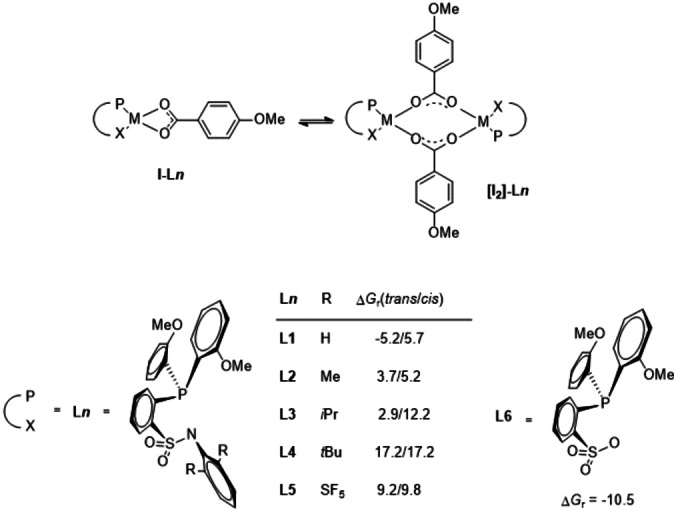
Relative Gibbs free reaction energies Δ*G*_r_ (kcal/mol) in dimerizations of various complexes of type **I**, with the atoms P and X of the two ligands being on opposite sides in the dimer (*trans*, as shown in the figure) or on the same sides (*cis*) to each other. While the computation of the *cis*‐isomer of **[I2]**‐**L1** was carried out mainly for completeness the one for **[I2]**‐**L6** was spared, as there is no reason to assume that an endergonic *cis*‐isomer would form as long as an exergonic *trans*‐alternative is available.

Carboxylate complexes **I**, which are the anticipated starting points of the catalytic cycle shown in Figure 1, tend to dimerize to very stable bis‐palladium‐bis‐carboxylato compounds of the type **[I_2_]**‐**L** (Figure 2). *Trans*‐ and *cis*‐forms of the dimers are conceivable depending on the relative positions of the P and X donors of the two ligands in the dimers. As a first step we therefore investigated how large the substituents at the periphery of the ligands would need to be to avoid such a dimerization. The results are shown in Figure 2.

From a synthetic viewpoint it is preferred to vary the substituents at the *ortho* positions of the aryl ring at the amido nitrogen atom. Describing the *trans*‐compounds first, it can be seen that complex **I**–**L1**, carrying H at these positions, will readily dimerize as the reaction free energy amounts to −5.2 kcal/mol. Substituting the H centers with Me‐, *i*Pr‐ and *t*Bu‐groups in **I**–**L2**, **I**–**L3** and **I**–**L4**, respectively, changes the situation as the reaction free energies of 3.7, 2.9 and 17.2 kcal/mol, respectively, indicate endergonic reactions. The *cis*‐forms of complexes **I**–**L1** to **I**–**L4** are all endergonic. This is valuable information for experimental work as it creates notable possibilities for variations in ligand design enabling experimentalists to choose between methyl, isopropyl and *tert*‐butyl substituents. Experimentally, we already could observe for **I**–**L3** the monomeric structure to exist both in solution as in the solid state in accordance with the computations. SF_5_‐groups in 2,6‐position of the sulfonamide phenyl group also are useful to prevent dimerization, as is indicated by the Gibbs free reaction energy of 9.2 and 9.8 kcal/mol for the *trans* and the *cis*‐isomer of **I**–**L5**. However, sulfonato complexes as **I**–**L6** that are very interesting with regard to the ES (see below) dimerize spontaneously as can be concluded from the Gibbs free reaction energy of −10.5 kcal/mol for **[I_2_]**‐**L6**. Going from **L1** to **L4** the increasing size of the substituents at the phenyl ring of the sulfonamido group leads to an elongation of the N−Pd as well as the P−Pd bonds, while the Pd−Pd distances also increases. As a result the overlap of the ligand donor orbitals with Pd orbitals is diminished, leading to a destabilization of the dimers which is pushed apart as the substituents grow larger (for a more detailed analysis see Supporting Information).

## 2. Variation of the substituents in the substrate

For the parent system (ligand **L3**, substrate anisol, see Figure 2) we have already computed an ES of 32.3 kcal/mol.^[9]^ This value rises to 37.7 kcal/mol upon substitution of the methoxy group of the anisolate by hydrogen. If instead of hydrogen a fluoride ligand is placed at the same position the ES amounts to 36.7 kcal/mol. This indicates that it is more beneficial with regard to the ES if substrates with electron donating substituents such as OMe groups are used.

This feature of the proposed catalytic cycle contrasts with the mechanism of the currently established catalytic system that rely largely on deprotonation of the aromatic C−H bonds. Thus, only substrates with the necessary C−H bond acidity can be carboxylated, similar to reactions involving strong bases.^[11]^ Overcoming this limitation on the substrate scope would greatly expand the synthetic utility of catalytic arene carboxylation.

## 3. Variations in the ligand

### 3.1. Other donor elements than nitrogen in the sulfonamido part of the ligand

To evaluate the influence of the donor group X, the N atom of **L3** was replaced by oxygen (**L6**), sulfur (**L7**), and carbon (**L8**). This leads to ES values of 28.3, 33.6 and 41.7 kcal/mol, respectively. While substitutions with sulfur and carbon do not lower the ES the opposite is true for oxygen, and the ES of 28.3 for **L6** represents a significant reduction of the ES relative to the parent system (32.3 kcal/mol) by 4.0 kcal/mol. However, upon replacing the SO_2_‐group of **L6** by a methylene unit as in **L9**, the ES increases to 33.6 kcal/mol. This shows that it is not only the metal bound oxygen atom in **L6** but the whole SO_2_ group is responsible for the reduction of the ES. As an overall result of this part of the study it can be stated that in this fragment of the ligand sulfonamido and sulfonato units generate the lowest ES values.


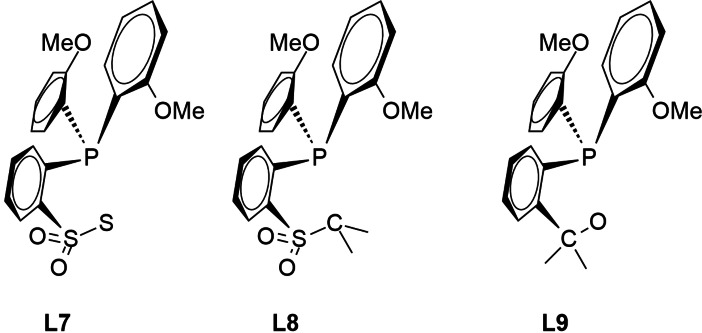


Upon replacement of one of the two oxygen atoms of the sulfonamido group by nitrogen, generating RS(O)(=NR)N groups the following results were obtained. The ES values of complexes with ligands **L10**, **L11** and **L12** amount to 33.2, 33.9 and 32.6 kcal/mol, respectively, and thus deviate only marginally from the ES of the parent systems (32.3 kcal/mol). The attempt to retain the 2,6‐dimethyl substitution at the phenyl ring at the N center of the sulfonamido group (prevention of dimerization) and at the same time lower the ES by modulating the electronic situation in close proximity of the sulfur atom does not generate the desired effect.


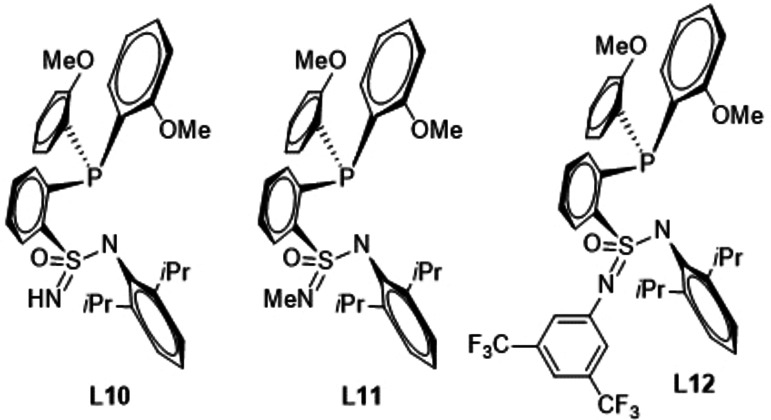


### 3.2. Variations of the phosphane part of the ligand

In the next step, the phosphane part of the ligand was varied. At first, the phosphorous atom was exchanged by nitrogen (**L13**). The computed ES value amounts to 38.7 kcal/mol and thus is significantly higher than the one of the parent system (32.3 kcal/mol) indicating that such a substitution was unfavorable.


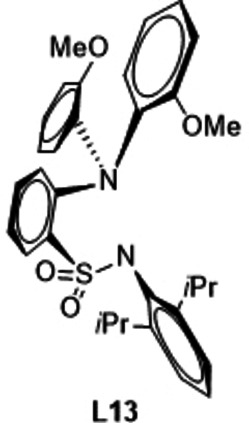


If the *ortho*‐anisyl substituents at the phosphorous center of ligand **L3** are substituted with additional OMe groups in 3,5‐position (**L14**) the ES value is reduced by 0.4 kcal/mol to 31.9 kcal/mol, while an analogous substitution by CF_3_ groups (**L15**) generates a very strongly increased ES of 57.3 kcal/mol. Therefore, electron‐withdrawing groups appear to be detrimental for the ES, while electron‐donating groups do lower the ES, even though this effect is not very pronounced.


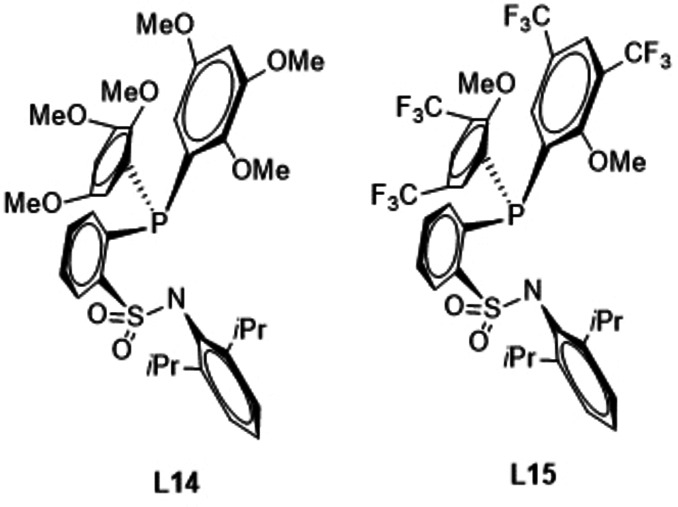


## 4. Combined variations in the substrate and in the ligand

The decrease of the ES is much more pronounced if the substrate has a second electron‐donating substituent. In **I**–**L3**‐**S1** for instance an additional OMe group is located at the *ortho* position of the OMe group already present, and the ES for this system amounts to 27.8 kcal/mol. This is a substantial decrease of the ES by 4.5 kcal/mol when compared with the ES of the parent system (32.3 kcal/mol). If additionally one further OMe group is introduced in the 4 position at both *ortho*‐anisyl substituents of the ligand relative to the P bound carbon centers of the aromatic rings, the ES decreases even more to 26.8 kcal/mol (**I**–**L16**‐**S1**). The introduction of an additional CF_3_ group at the phenyl ring of the sulfonamido part of the ligand (in *para* position relative to the N bound carbon center of the aromatic ring) increases the ES slightly to 27.6 kcal/mol (**I**–**L17**‐**S1**). It is interesting to note, that the introduction of an OMe group in the 4 position of the phenyl ring in the sulfonamide part of the ligand also increases the ES slightly to 28.3 kcal/mol (**I**–**L18**‐**S1**). This means that independent of the electronic properties of the substituent (CF_3_ in **I**–**L17**‐**S1**; OMe in **I**–**L18**‐**S1**) the ES rises slightly relative to the otherwise analogous system in **I**–**L16**‐**S1**.


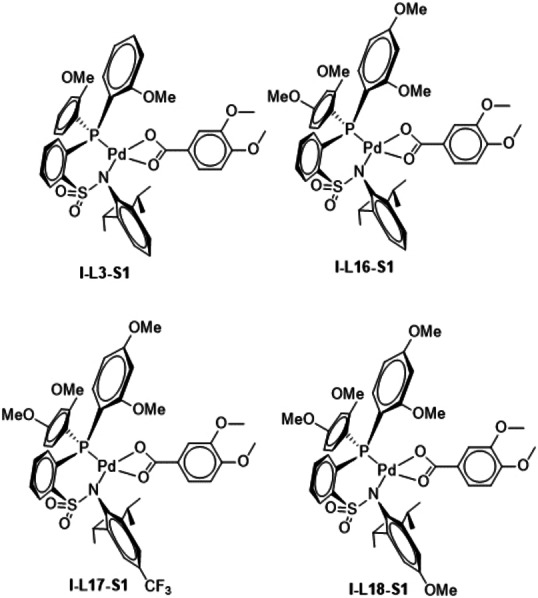


### 4.1. Conclusions from the ligand/substrate screening

The most important results of this screening can be summarized as follows:

1. Because of the remarkably low ES of 28.3 kcal/mol relative to the parent system a change from sulfonamido ligands to sulfonato ligands appears to be most beneficial. Further work to explore the structural variation to simultaneously prevent any tendency for dimerization with this ligand framework seems therefore promising.^[12]^
2. The variations of the substituents in the sulfonamido part of the ligand do not lead to very pronounced changes in ES values and would be therefore less relevant to optimize an experimental system efficiently.
3. Electron‐withdrawing substituents in the phosphane part of the ligand are disadvantageous, while electron donating substituents are beneficial. Again, this influence is comparably low, however.
4. Electron‐withdrawing substituents in the substrate are not advantageous for the reaction while electron‐donating substituents are beneficial and to some extent additive. Thus, the mechanistic principle holds potential for the carboxylation to arenes that are not C−H acidic.

## 5. Influence of the base

**Figure 3:**
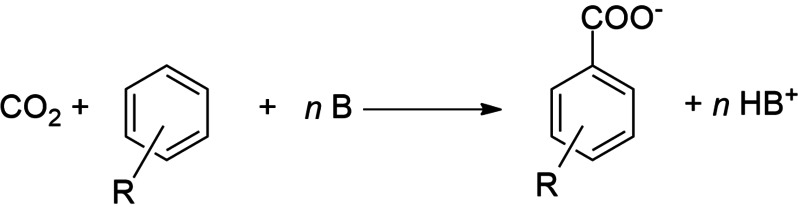
CO_2_ insertion into an arene and concomitant stabilization of the carbonic acid with a Bronstedt base.

In the next step, the influence of the base is investigated. As the formation of carbonic acids from CO_2_ and arenes is endergonic, a base is needed to generate an exergonic end product in an acid‐base reaction (Figure 3). After having calculated quite a variety of adducts using amine bases (see Supporting Information) it occurred to us that it might not be possible to generate a clearly exergonic product, i.e. amine bases like NEt_3_ and HN(*i*Pr)_2_ might be too weak. Upon switching to potassium *tert*‐butoxide as the base a Gibbs free reaction energy of −13.4 kcal/mol was computed for a 1 : 1‐adduct indicating strong alcoholate bases to be the better choice over amine bases in experimental work.

## 6. Analysis of the complete catalytic cycle for Pd and Pt catalysts

**Figure 4:**
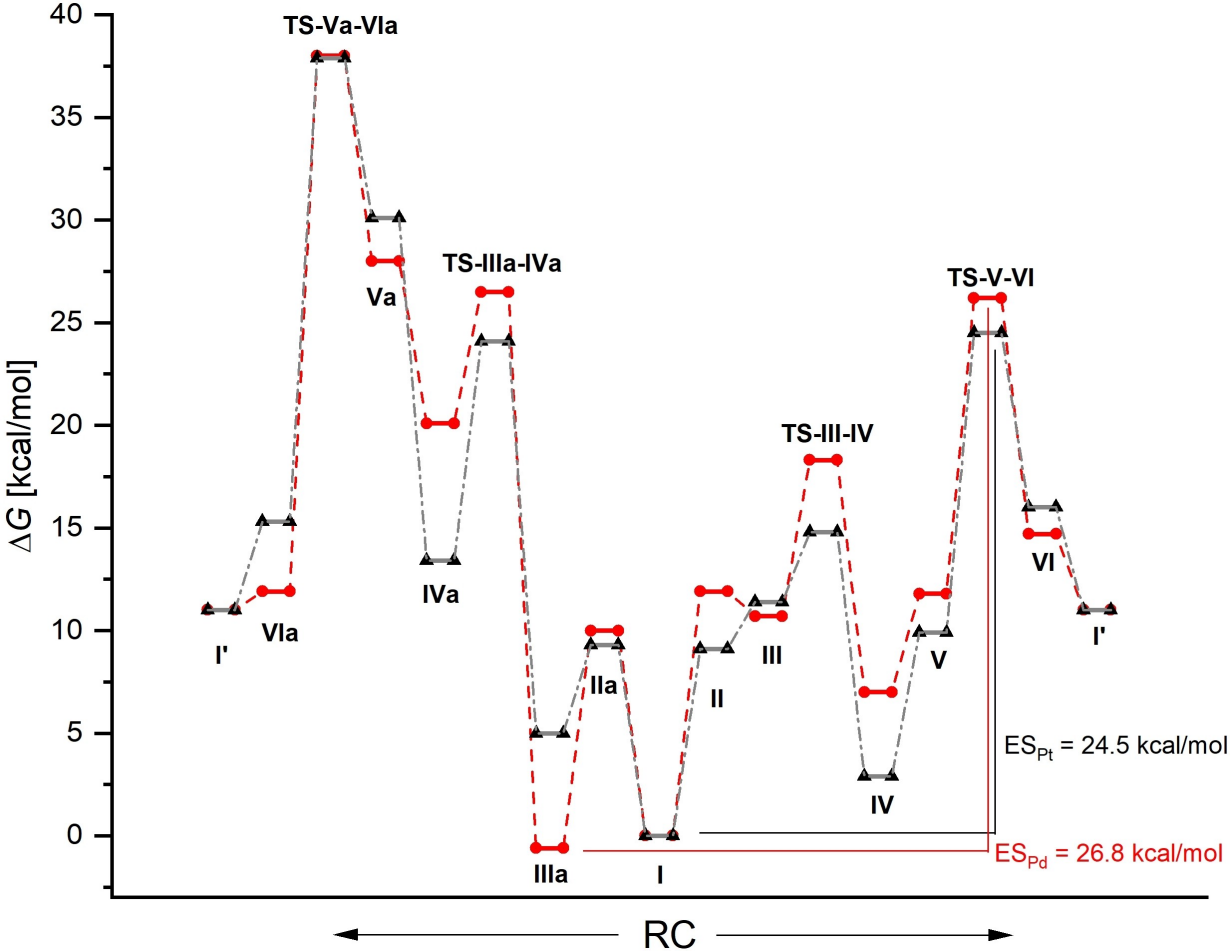
Computed energy profiles for the catalytic cycles shown in Figure 1 for complexes **I(Pd)**‐**L16**‐**S1** (red) and **I(Pt)**‐**L16**‐**S1** (black) in dichloromethane solution (B97‐D3BJ/def2‐TZVP(ECP)/SMD) with relative Gibbs free energies Δ*G* (kcal/mol) and indications of the TDI and TDTS as well as ES values for both systems.

Based on the results for the individual components of the catalytic system in sections 1–5, the most promising Pd complex **I(Pd)**‐**L16**‐**S1** was selected to compute and analyse a complete catalytic cycle as shown in Figure 1. In order to evaluate the influence of the metal in the same ligand framework, the cycle was analysed also for the analogous complex of the corresponding 5d metal platinum. The resulting energy profiles for complexes **I(Pd)**‐**L16**‐**S1** and **I(Pt)**‐**L16**‐**S1**are shown in Figure 4.

All relevant local minima and transition states as shown in Figure 1 could be localized for both catalyst systems. For both metals the reaction pathway in which the carboxylate in **I** opens towards the N center of the sulfonamide group is energetically much lower in comparison to the alternative pathway where the carboxylate in **I** opens towards the phosphane part of the ligand. The energetically more unfavorable pathway leads to ES values for the Pd and the Pt system of 38.0 and 37.9 kcal/mol, respectively. For the lower energy pathway, the transition state **TS**‐**V**–**VI** is the TDTS for both metals. For the Pt compound reference point **I** is the TDI, while in the case of Pd a slightly more stable minimum **IIIa** is the TDI. This leads to ES values of 26.8 kcal/mol and 24.5 kcal/mol for the Pd and the Pt system, respectively. To obtain an impression if various density functionals would scatter substantially in this regard, we recomputed structures **I** and **TS**‐**V**–**VI** for both the Pd and the Pt system using B3LYP−D3BJ, MN15‐L and PBE0‐D3BJ while maintaining the basis set and compared the computed values with the ones obtained before. The energy differences for those DFs deviate from the ones obtained with B97‐D3BJ within an acceptably narrow range of a few kilocalories per mole and therefore the overall picture does not change (details see Supporting Information). To obtain a strongly simplified though very illustrative first impression of the rate of the reaction one can use the ES as the activation barrier in the Eyring equation and assume that the calculated rate constant resembles the TOF of the reaction. At a reaction temperature of 80 °C TOFs of 0.7 and 18 h^−1^ are computed for the Pd and the Pt system in dichloromethane solutions. In other words: these complexes may offer potential lead structures for productive turnover if the proposed catalytic cycle is not interrupted by undesired side reactions. In order to assess such deactivation pathways, several alternative structures and reactivity patterns were assessed also.

### 6.1. Undesired side reactions

A possible interference with the desired catalytic cycle would result if the substrate 1,2‐dimethoxy benzene coordinates via the oxygen atom of the OMe group as in **VII** and **VIIa** instead of coordinating via the C−H bond as in **II** and **IIa**. This could also be the case for **V** and **Va** in which an oxygen coordination by the substrate would yield complexes **VIII** and **VIIIa**. Gratifyingly, the computations showed **VII**/**VIIa** as well as **VIII**/**VIIIa** to be all endergonic for both metals relative to the reference point **I** indicating that these compounds do not contribute disadvantageously to the energy span.


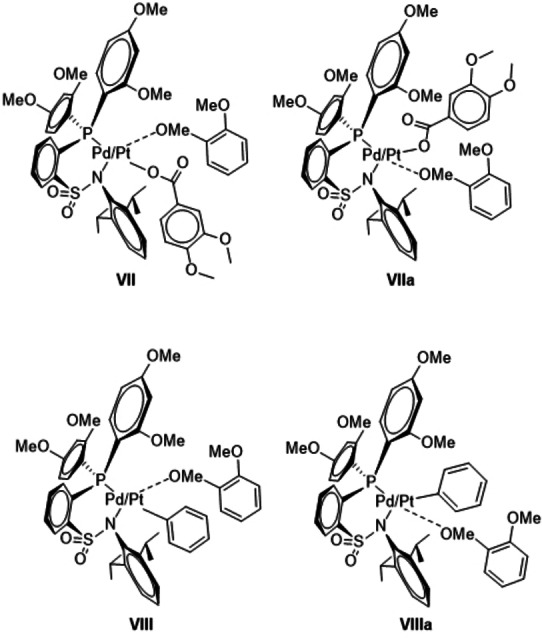


Furthermore, after a first successful catalytic cycle it could in principal be possible that the produced acid protonates the sulfonamide N center generating complexes **IX**. Again, the computations showed such complexes to be endergonic relative to the reference point, and therefore the ES will not be changed if a species like **IX** would be formed.


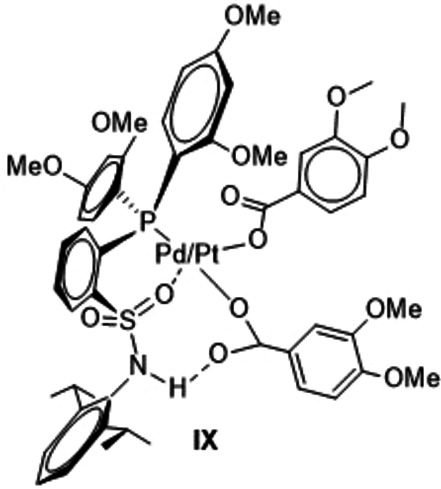


## 7. Summary and Conclusions

In this work we studied by means of DFT computations the potential of Pd and Pt complexes bearing bidentate phosphano‐sulfonamido‐complexes as catalysts for the catalytic carboxylation of arenes. All components of a possible catalytic system were included in the analysis. A variety of ligands as well as concrete Pd and Pt complexes were generated in silico and additional conditions such as dimerization of carboxylate complexes **I**, the nature of the substrate, the choice of base and undesired side reactions were considered. The sulfonamido part of the general ligand system employed in this study is well suited to create the necessary steric bulk, which prevents a dimerization of the active starting species **I** of a potential catalytic cycle. The introduction of electron‐donating substituents at the phenyl groups in the phospane part of the catalyst helps to decrease the ES to some extent. The most pronounced decrease of the ES was found to result from a) electron‐donating substituents in the substrate and b) changing the catalyst metal from palladium to platinum. For the most favorable combinations, ES values in the mid 20 kcal/mol regime were calculated opening the door to productive turnover from an energy point of view, provided the catalysts exhibit a decent thermal stability. Obvious undesired side reactions, such as non‐productive types of substrate coordination, appear not to be prohibitive as they do not alter the ES in a disadvantageous manner. With regard to the overall required exergonicity of the net reaction the computations suggest simple amine bases to be most likely too weak to generate a negative Gibbs free reaction energy for the formation of aromatic carboxylate salts. This can be overcome, however, by the use of bulky alcoholate bases. Alternatively, more polar reaction media might also be envisaged in future studies.

While purely predictive computational studies have the inherent limitation of not being able to cover every possible side reaction or deactivation pathway that might occur under the real operating conditions, the energetic balance of proposed pathways can be assessed with sufficient accuracy^[13]^ to provide guidelines for directions to be experimentally verified. In this sense, the overall encouraging results of this study do suggest that it is worthwhile to investigate such catalyst systems as presented here experimentally. In addition to the corresponding efforts ongoing in our laboratories, we will continue to explore the general principle of arene carboxylation via the coupled sequence of CO_2_ insertion and C−H activation computationally using the.systems approach described herein.

## Conflict of interest

The authors declare no conflict of interest.

## Supporting information

As a service to our authors and readers, this journal provides supporting information supplied by the authors. Such materials are peer reviewed and may be re‐organized for online delivery, but are not copy‐edited or typeset. Technical support issues arising from supporting information (other than missing files) should be addressed to the authors.

Supporting Information

Supporting Information

## Data Availability

The data that support the findings of this study are available in the supplementary material of this article.
