# Supplementary material for: Factors Governing the Catalytic Insertion of CO2 into Arenes – A DFT Case Study for Pd and Pt Phosphane Sulfonamido Complexes
Source: Chemistry. 2022 Mar 21;28(23):e202104375. doi: 10.1002/chem.202104375 (PMC9310616; doi:10.1002/chem.202104375)
Supplement: Supplementary file 1 — Supporting Information [file CHEM-28-0-s001.pdf]

Chemistry–A European Journal

Supporting Information

Factors Governing the Catalytic Insertion of CO_2 into Arenes – A DFT Case Study for Pd and Pt Phosphane Sulfonamido Complexes

Markus Hölscher,* Gregor Kemper, Sangeth Jenthra, Carsten Bolm, and Walter Leitner*

Author Contributions

M.H. Conceptualization:Lead; Investigation:Lead; Writing – original draft:Lead; Writing – review & editing:Equal
G.K. Data curation:Supporting; Writing – review & editing:Supporting
S.J. Data curation:Supporting; Writing – review & editing:Supporting
C.B. Conceptualization:Supporting; Investigation:Supporting; Writing – review & editing:Equal
W.L. Conceptualization:Supporting; Investigation:Supporting; Writing – review & editing:Equal

Computational details

The DFT-calculations in this work were performed using the GAUSSIAN *16 suite of programs (Revision B.01)*[1]. With some exceptions (see below) the B97[2] density functional was employed in this work together with the Grimme D3-dispersion correction augmented with the Becke-Johnson damping (B97-D3(BJ))[3], with the automatic density fitting approximation switched on (set to auto).

For geometry optimizations concerning the monomer/dimer section of the main paper (section 1; Table S1 of this SI) the def2-SVP[4] basis set was applied with the corresponding ECP[5] for the metal centre(s). After optimization a frequency calculation was run to ensure the existence of local minima (*i* = 0). Those structures were then used for single-point-energy-calculations using the def2-TZVP basis set[4] employing the corresponding ECP for the metal centre(s)[5]. The higher level electronic energies $E_{(\mathrm{TZVP})}$ from these single point calculations were then added to the thermal correction term obtained from the lower level geometry optimization [$G_{(SVP)}$-$E_{(SVP)}$] and in this way the relative Gibbs free energy differences [$\Delta G\mathrm{rel}_{(\mathrm{TZVP})}$] were computed. Implicit solvent corrections were applied by using the SMD solvation model[6] and dichloromethane as the solvent both in the low level geometry optimization and in the high level single point calculation. To account for standard state conditions a pressure of 383 atm was applied[7].

For all other compounds in this work (Tables S2 to S4) optimizations were run on B97D3-BJ/def2-TZVP(ECP)/SMD[2-6] also applying implicit solvent corrections for DCM. To account for standard state conditions a pressure of 383 atm was applied[7].In this way all compounds were at first optimized and subsequently input to frequency calculations to characterize the nature of the stationary points to be a minimum (*i*=0) or a transition state (*i*=1). IRC calculations were carried out for some compounds to verify that the localized transition state indeed does connect the independently localized preceding/following minima.

Finally, all compounds necessary to compute the energy difference between I and TS-V-VI mentioned in Table S4, were recomputed using various density functionals, namely, B3LYP-D3BJ, MN15-L, PBE0-D3BJ to evaluate the influence of the used density functional on that energy difference (see Table S5).

Optimized structures will be made available als mol-files in a separate file.

Table S1: Computed energies (*E*), zero point energy corrected energies (*E*zpe), enthalpies (*H*) and Gibbs free energies (*G*; all in Hartree, all optimized on B97D3-BJ/def2-SVP/SMD/dichloromethane) for monomers and dimers as shown in Figure 2 of the main paper together with single point energies [*E*(TZVP), computed on B97D3/def2-TZVP/SMD/dichloromethane] and relative Gibbs free energy differences [Δ*G*rel(TZVP); in kcal/mol].

Compd.	*E*	*E*zpe	*H*	*G*	*G*-*E*	*E*(TZVP)	*E*(TZVP)+(*G*-*E*) = *G*(TZVP)	ΔGrel(TZVP)
**I-L1**	-2760.073648	-2759.510815	-2759.467233	-2759.584896	0.4887521	-2762.445554	-2761.956802	0.0
trans-**[I_2]-L1**	-5520.207122	-5519.078258	-5518.991628	-5519.199019	1.0081033	-5524.930024	-5523.921921	-5.2
cis-**[I_2]-L1**	-5520.189273	-5519.060015	-5518.973506	-5519.179663	1.0096102	-5524.914198	-5523.904588	5.7
**I-L2**	-2838.609188	-2837.992663	-2837.945712	-2838.070143	0.5390452	-2841.061857	-2840.522811	0.0
trans-**[I_2]-L2**	-5677.270282	-5676.032062	-5675.939386	-5676.156476	1.1138059	-5682.153549	-5681.039744	3.7
cis-**[I_2]-L2**	-5677.264887	-5676.027758	-5675.93473	-5676.152898	1.111989	-5682.149356	-5681.037367	5.2
**I-L3**	-2995.66203	-2994.934072	-2994.881912	-2995.016663	0.6453668	-2998.275101	-2997.629735	0.0
trans-**[I_2]-L3**	-5991.372489	-5989.911527	-5989.808267	-5990.046188	1.3263008	-5996.58114	-5995.25484	2.9
cis-**[I_2]-L3**	-5991.361979	-5989.900795	-5989.797674	-5990.03437	1.3276094	-5996.567702	-5995.240092	12.2
**I-L4**	-3074.160675	-3073.37737	-3073.322997	-3073.4605	0.700175	-3076.853635	-3076.15346	0.0
trans-**[I_2]-L4**	-6148.346721	-6146.776393	-6146.66818	-6146.912509	1.434212	-6153.713754	-6152.279542	17.2
cis-**[I_2]-L4**	-6148.350603	-6146.778455	-6146.671058	-6146.91318	1.4374227	-6153.716913	-6152.27949	17.2
**I-L5**	-4551.980011	-4551.403826	-4551.348364	-4551.489547	0.4904635	-4555.937454	-4555.446991	0.0
trans-**[I_2]-L5**	-9104.006241	-9102.85049	-9102.740248	-9102.992246	1.0139949	-9111.893213	-9110.879218	9.2
cis-**[I_2]-L5**	-9104.009144	-9102.852609	-9102.74271	-9102.993591	1.0155529	-9111.893963	-9110.87841	9.8
**I-L6**	-2549.179313	-2548.707172	-2548.668599	-2548.774409	0.4049036	-2551.349299	-2550.944395	0.0
trans-**[I_2]-L6**	-5098.420179	-5097.47253	-5097.39546	-5097.584833	0.8353458	-5102.740933	-5101.905587	-10.5

Table S2: Computed energies (E), zero point energy corrected energies (Ezpe), enthalpies (H) and Gibbs free energies (G; all in Hartree, all optimized on B97D3-BJ/def2-TZVP/SMD/dichloromethane) for optimzed TDIs (complexes **I**) and TDTS (complexes **TSV-VI**) as discussed in sections 2-4 of the main paper. Relative Gibbs free energy differences between TDI and TDTS are given as well (ΔGrel; in kcal/mol).[a]

Ligand	Compd.	*E*	*E*zpe	*H*	*G*	ΔGrel
	p-Anisic acid	-535.2484259	-535.104057	-535.093236	-535.134054	
	Anisol	-346.6899911	-346.560253	-346.55224	-346.585894	
	CO2	-188.5621082	-188.5508	-188.54721	-188.566552	
	3,4-Dimethoxy-benzoicacid	-649.7488	-649.5729	-649.5594	-649.60666	
	1,2-Dimethoxybenzene	-461.1899	-461.0285	-461.0179	-461.057572	
**L6**	**I**	-2551.355163	-2550.884327	-2550.845604	-2550.95282	
	**TSV-VI**	-2551.330737	-2550.86176	-2550.823352	-2550.92618	28.3
**L7**	**I**	-2874.37562	-2873.907308	-2873.867751	-2873.97772	
	**TSV-VI**	-2874.341495	-2873.875464	-2873.836064	-2873.94249	33.6
**L8**	**I**	-2594.031787	-2593.484499	-2593.442057	-2593.55767	
	**TSV-VI**	-2593.986422	-2593.441473	-2593.399299	-2593.50953	41.7
**L9**	**I**	-2120.582775	-2120.041074	-2120.001305	-2120.109552	
	**TSV-VI**	-2120.548813	-2120.009149	-2119.969643	-2120.074312	33.6
**L10**	**I**	-2978.368636	-2977.632386	-2977.57926	-2977.71668	
	**TSV-VI**	-2978.33643	-2977.602491	-2977.549604	-2977.68224	33.2
**L11**	**I**	-3017.659029	-3016.895504	-3016.840427	-3016.98363	
	**TSV-VI**	-3017.626852	-3016.865594	-3016.8109	-3016.94799	33.9
**L12**	**I**	-3883.406668	-3882.583212	-3882.517724	-3882.68742	
	**TSV-VI**	-3883.375454	-3882.55446	-3882.48923	-3882.65388	32.6
**L13**	**I**	-2711.57768	-2710.84736	-2710.796014	-2710.927651	
	**TSV-VI**	-2711.536769	-2710.808216	-2710.757304	-2710.884436	38.7
**L14**	**I**	-3456.289226	-3455.436371	-3455.373072	-3455.531712	
	**TSV-VI**	-3456.258233	-3455.408049	-3455.344881	-3455.499282	31.9
**L15**	**I**	-4346.360022	-4345.617895	-4345.550384	-4345.72257	
	**TSV-VI**	-4346.283829	-4345.542528	-4345.475183	-4345.64967	57.3
**L3-S1**	**I**	-3112,778315	-3112,021112	-3111,965451	-3112,10965	
	**TSV-VI**	-3112,755392	-3112,000178	-3111,944987	-3112,08375	27.8
**L16-S1**	**I**	-3341.793149	-3340.971927	-3340.91123	-3341.06536	
	**TSV-VI**	-3341.770882	-3340.952008	-3340.891551	-3341.04107	26.2
**L17-S1**	**I**	-3678.821328	-3677.996751	-3677.931865	-3678.09882	
	**TSV-VI**	-3678.799891	-3677.977471	-3677.912999	-3678.07323	28.3
**L18-S1**	**I**	-3456.29914	-3455.446816	-3455.383176	-3455.54427	
	**TSV-VI**	-3456.276046	-3455.425471	-3455.362324	-3455.51751	27.8

[a] The relative Gibbs free energie differences ΔGrel were computed as follows: To the energy of the reference point (intermediate **I**) were added the energies of all other reactants (i.e. one CO_2 and one arene molecule). From this value the sum of the energies of **TS V-VI** and one carbonic acid molecule were subtracted.

Table S3: Computed energies (*E*), zero point energy corrected energies (*E*zpe), enthalpies (*H*) and Gibbs free energies (*G*; all in Hartree, all optimized on B97D3-BJ/def2-TZVP/SMD/dichloromethane) for the acid base reation as shown in Figure 3 of the main paper. Relative Gibbs free reaction energies are given as well (Δ*G*rel; in kcal/mol).

Compds.	*E*	*E*zpe	*H*	*G*	Δ*G*rel
**Bases**					
Triethylamine	-292.3459431	-292.145074	-292.134587	-292.173351	
Diisopropylamine	-292.3571066	-292.156432	-292.146000	-292.184208	
Potassium-*tert*-butoxide	-833.0611730	-832.941418	-832.931921	-832.970139	
**Reactants**					
Anisol	-346.689991	-346.560253	-346.55224	-346.585894	
Benzene	-232.185014	-232.086836	-232.081396	-232.108732	
Trifluoromethylbenzene	-569.2110083	-569.108768	-569.099717	-569.138201	
1,3-di(trifluoromethyl)benzene	-906.234637	-906.128416	-906.115677	-906.163489	
CO2	-188.562108	-188.550800	-188.547210	-188.566552	
**Products**[a]					
**A**	-827.618295	-827.270257	-827.248544	-827.316334	5.9
**B**	-827.630546	-827.283125	-827.261382	-827.329233	4.7
**C**	-1120.00926	-1119.458695	-1119.42589	-1119.51800	1.8
**D**	-1005.50347	-1004.98469	-1004.954423	-1005.04159	1.3
**E**	-1342.53026	-1342.00740	-1341.973423	-1342.06998	2.0
**F**	-1679.55541	-1679.02852	-1678.990729	-1679.09868	-0.1
**G**	-2016.55391	-2016.023169	-2015.981999	-2016.09439	-0.7
**H**	-1134.71357	-1134.580987	-1134.568196	-1134.61604	-13.4
tert-butanol	-233.636898	-233.504573	-233.496835	-233.527969	

[a] For a correlation of compound numbers **A** to **H** with their structures see Figure S1 below.

Figure S1: Gibbs free reaction energies according to Figure 3 of the main paper and Table S3 (above) showing various adducts of the anion of the carbonic acid with protonated amine bases in various compositions as well as the adduct formed from the reaction using potassium-*tert*-butoxide. For *n* = 2 hydrogen bonds form between the acid and both base molecules.

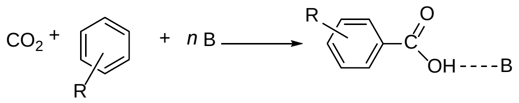

Compd.	R on arene	Base	*n*	Δ*G*r
**A**	OMe	NEt3	1	5.9
**B**	OMe	HN(*i*Pr)2	1	4.7
**C**	OMe	HN(*i*Pr)2	2	1.8
**D**	H	HN(*i*Pr)2	2	1.3
**E**	CF3	HN(*i*Pr)2	2	2.0
**F**	1,3-(CF3)2	HN(*i*Pr)2	2	-0.1
**G**	1,2,3-(CF3)3	HN(*i*Pr)2	2	-0.7
**H**	OMe	K(*t*BuO)	1	-13.4

Table S4: Computed energies (*E*), zero point energy corrected energies (*E*zpe), enthalpies (*H*) and Gibbs free energies (*G*; all in Hartree, all optimized on B97D3-BJ/def2-TZVP/SMD/dichloromethane) computed for the catalytic cycles according to Figure 1 of the main paper and directly related to the energy profiles of the Pd and Pt complexes **I(Pd)-L16-S1** and **I(Pt)-L16-S1**, respectively, shown in Figure 4 of the main paper. Relative Gibbs free reaction energies are given as well (Δ*G*rel; in kcal/mol).

Compd...	*E*	*Ezpe*	*H*	*G*	Δ*G*rel
Palladium system					
**I**	-3341.793149	-3340.971927	-3340.91123	-3341.065361	0.0
**II**	-4301.577832	-4300.727022	-4300.660811	-4300.827818	11.9
**III**	-3802.984558	-3802.000704	-3801.928444	-3802.105803	10.7
**IV**	-3802.992266	-3802.007517	-3801.935895	-3802.111818	7.0
**V**	-3341.789242	-3340.97021	-3340.908295	-3341.064004	11.8
**VI**	-3341.792919	-3340.971211	-3340.911022	-3341.059327	14.7
**TSIII-IV**	-3802.970471	-3801.990828	-3801.919764	-3802.093754	18.3
**TSV-VI**	-3341.770882	-3340.952008	-3340.891551	-3341.041066	26.2
**IIa**	-4301.588199	-4300.736820	-4300.670993	-4300.835438	7.1
**IIIa**	-3802.997259	-3802.013878	-3801.941128	-3802.123882	-0.6
**IVa**	-3802.971122	-3801.986717	-3801.915163	-3802.090904	20.1
**Va**	-3341.762492	-3340.943887	-3340.881915	-3341.03827	28.0
**VIa**	-3341.796113	-3340.974194	-3340.91396	-3341.063843	11.9
**TSIIIa-IVa**	-3802.95623	-3801.976329	-3801.905113	-3802.0807	26.5
**TSVa-VIa**	-3341.750046	-3340.931227	-3340.870672	-3341.022277	38.0
Platinum system					
**I**	-3333.245224	-3332.424094	-3332.363294	-3332.518631	0.0
**II**	-4293.036665	-4292.185141	-4292.119335	-4292.285497	9.1
**III**	-3794.440674	-3793.455249	-3793.383924	-3793.558023	11.4
**IV**	-3794.453556	-3793.468585	-3793.397148	-3793.571624	2.9
**V**	-3333.245692	-3332.426326	-3332.364516	-3332.520308	9.9
**VI**	-3333.244077	-3332.422089	-3332.361929	-3332.510681	16.0
**TSIII-IV**	-3794.429818	-3793.449925	-3793.379014	-3793.552535	14.8
**TSV-VI**	-3333.227049	-3332.407401	-3332.347147	-3332.497021	24.5
**IIa**	-4293.040134	-4292.187932	-4292.122471	-4292.285245	9.3
**IIIa**	-3794.444679	-3793.460563	-3793.388189	-3793.568299	5.0
**IVa**	-3794.436057	-3793.451349	-3793.379894	-3793.554919	13.4
**Va**	-3333.213564	-3332.394295	-3332.332607	-3332.488126	30.1
**VIa**	-3333.245117	-3332.422667	-3332.362585	-3332.511789	15.3
**TSIIIa-IVa**	-3794.413764	-3793.4345	-3793.363312	-3793.537735	24.1
**TSVa-VIa**	-3333.205373	-3332.385956	-3332.325682	-3332.475739	37.9
CO2	-188.562108	-188.5508	-188.54721	-188.566552	
CH2Cl2	-959.788788	-959.760128	-959.755538	-959.781382	
3,4-dimethoxy-benzoicacid	-649.748774	-649.572926	-649.559365	-649.60666	
1,2-dimethoxybenzene	-461.189925	-461.028465	-461.017862	-461.057572	

Table S5: Computed energies (*E*), zero point energy corrected energies (*E*zpe), enthalpies (*H*) and Gibbs free energies (*G*; all in Hartree) computed using various density functionals for stationary points **I** and **TS-V-VI** as shown in Figures 1 and 4 of the main paper. Relative Gibbs free reaction energies are given as well (ΔGrel; in kcal/mol).[a]

DF/Metal/Compd...	*E*	*Ezpe*	*H*	*G*	ΔGrel
B3LYP-D3BJ					
CO2	-188.669332	-188.657719	-188.654154	-188.673449	
1,2-dimethoxybenzene	-461.4975528	-461.332646	-461.322238	-461.361696	
3,4-dimethoxy-benzoicacid	-650.1669665	-649.98731	-649.974016	-650.020766	
Pd					
**I**	-3343.197542	-3342.358481	-3342.299169	-3342.45053	0.0
**TS-V-VI**	-3343.162811	-3342.326462	-3342.267227	-3342.414912	31.4
Pt					
**I**	-3334.655791	-3333.816282	-3333.757023	-3333.908785	0.0
**TS-V-VI**	-3334.62545	-3333.788319	-3333.729307	-3333.876782	29.1
MN15-L					
CO2	-188.5114049	-188.499602	-188.496044	-188.515337	
1,2-dimethoxybenzene	-461.0557569	-460.890318	-460.880135	-460.918888	
3,4-dimethoxy-benzoicacid	-649.5666288	-649.385982	-649.372921	-649.418945	
Pd					
**I**	-3340.254013	-3339.415809	-3339.357086	-3339.505172	0.0
**TS-V-VI**	-3340.232565	-3339.395195	-3339.337735	-3339.477241	27.1
Pt					
**I**	-3331.764808	-3330.925123	-3330.866671	-3331.014913	
**TS-V-VI**	-3331.747198	-3330.910261	-3330.852658	-3330.99299	0.0
					23.2
**PBE0-D3BJ**					
CO2	-188.4657124	-188.453904	-188.450348	-188.469625	
1,2-dimethoxybenzene	-460.9494247	-460.783513	-460.773194	-460.812313	
3,4-dimethoxy-benzoicacid	-649.4222786	-649.241267	-649.228064	-649.274517	
Pd					
**I**	-3339.945577	-3339.102652	-3339.043192	-3339.196183	0.0
**TS-V-VI**	-3339.909496	-3339.068595	-3339.009634	-3339.156748	29.4
Pt					
**I**	-3331.411288	-3330.567609	-3330.508361	-3330.660449	0.0
**TS-V-VI**	-3331.379269	-3330.538115	-3330.479178	-3330.626713	25.8

[a] For comparison note the values for the energy difference between **I** and **TS-V-VI** obtained with B97D3-BJ (see Table S4) for the Pd system and the Pt system to be 26.2 and 24.5 kcal/mol, respectively.

Pd-dimers

With regard to the discussion of dimer stabilities shown in Figure 2 of the main paper it can be noted, that the increasing steric bulk of the substituents at the phenyl ring of the sulfonamido N atom leads to an increasing demand of space. This is shown exemplarily in Figure S2 and Table S6 for Ligands **L1** to **L4**. While not strictly notable for each and every single bond length reported in Table S6 the general trend is clear, as it can be seen that the ligand donor atoms are located increasingly distant from the associated Pd centre as the ligand size/bulk increases. As indicated by the Pd-Pd distance the demand for space also pushes the two dimer units increasingly away. It is therefore plausible to assume that both the repulsive interactions between various molecular entities increase and also the interaction between the ligand donor atoms (P and N) with the Pd centres matches less well as the substituents grow larger.

Figure S2. Schematic representation of Pd-dimers for illustration of atom distances given in Table S6.

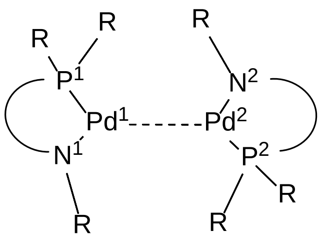

Table S6. Atom distances [A] for Pd-dimers as shown in Figure 2 of the main text.

Ligand	P1-Pd1	N1-Pd1	P2-Pd2	N2-Pd2	Pd1-Pd2
L1	2.229	2.070	2.244	2.048	4.473
L2	2.229	2.052	2.257	2.072	4.536
L3	2.239	2.060	2.248	2.083	4.723
L4	2.264	2.120	2.264	2.120	4.817

References:

[1] Gaussian 16, Revision B.01, M. J. Frisch, G. W. Trucks, H. B. Schlegel, G. E. Scuseria, M. A. Robb, J. R. Cheeseman, G. Scalmani, V. Barone, G. A. Petersson, H. Nakatsuji, X. Li, M. Caricato, A. V. Marenich, J. Bloino, B. G. Janesko, R. Gomperts, B. Mennucci, H. P. Hratchian, J. V. Ortiz, A. F. Izmaylov, J. L. Sonnenberg, D. Williams-Young, F. Ding, F. Lipparini, F. Egidi, J. Goings, B. Peng, A. Petrone, T. Henderson, D. Ranasinghe, V. G. Zakrzewski, J. Gao, N. Rega, G. Zheng, W. Liang, M. Hada, M. Ehara, K. Toyota, R. Fukuda, J. Hasegawa, M. Ishida, T. Nakajima, Y. Honda, O. Kitao, H. Nakai, T. Vreven, K. Throssell, J. A. Montgomery, Jr., J. E. Peralta, F. Ogliaro, M. J. Bearpark, J. J. Heyd, E. N. Brothers, K. N. Kudin, V. N. Staroverov, T. A. Keith, R. Kobayashi, J. Normand, K. Raghavachari, A. P. Rendell, J. C. Burant, S. S. Iyengar, J. Tomasi, M. Cossi, J. M. Millam, M. Klene, C. Adamo, R. Cammi, J. W. Ochterski, R. L. Martin, K. Morokuma, O. Farkas, J. B. Foresman, and D. J. Fox, Gaussian, Inc., Wallingford CT, 2016.

[2] A. D. Becke, *J. Chem. Phys.* **1997**, *107*, 8554-8560.

[3] a) S. Grimme, *Journal of computational chemistry* **2004**, *25*, 1463-1473; b) J. Antony, S. Grimme, *Physical Chemistry Chemical Physics* **2006**, *8*, 5287-5293; c) S. Grimme, *Journal of computational chemistry* **2006**, *27*, 1787-1799; d) M. Piacenza, I. Hyla-Kryspin, S. Grimme, *Journal of computational chemistry* **2007**, *28*, 2275-2285; e) S. Grimme, J. Antony, S. Ehrlich, H. Krieg, *The Journal of chemical physics* **2010**, *132*, 154104; f) S. Grimme, S. Ehrlich, L. Goerigk, *Journal of computational chemistry* **2011**, *32*, 1456-1465.

[4] a) A. Schäfer, H. Horn, R. Ahlrichs, *The Journal of Chemical Physics* **1992**, *97*, 2571-2577; b) A. Schäfer, C. Huber, R. Ahlrichs, *The Journal of Chemical Physics* **1994**, *100*, 5829-5835; c) K. Eichkorn, F. Weigend, O. Treutler, R. Ahlrichs, *Theoretical Chemistry Accounts* **1997**, *97*, 119-124; d) F. Weigend, F. Furche, R. Ahlrichs, *The Journal of chemical physics* **2003**, *119*, 12753-12762; e) F. Weigend, R. Ahlrichs, *Physical Chemistry Chemical Physics* **2005**, *7*, 3297-3305.

[5] a) D. Andrae, U. Haeussermann, M. Dolg, H. Stoll, H. Preuss, *Theoretica chimica acta* **1990**, *77*, 123-141; b) B. Metz, H. Stoll, M. Dolg, *The Journal of Chemical Physics* **2000**, *113*, 2563-2569.

[6] a) E. Cances, B. Mennucci, J. Tomasi, *The Journal of chemical physics* **1997**, *107*, 3032-3041; b) J. Tomasi, B. Mennucci, R. Cammi, *Chemical reviews* **2005**, *105*, 2999-3094; c) A. V. Marenich, C. J. Cramer, D. G. Truhlar, *The Journal of Physical Chemistry B* **2009**, *113*, 6378-6396.

[7] R. L. Martin, P. J. Hay, L. R. Pratt, *The Journal of Physical Chemistry A* **1998**, *102*, 3565-3573
